# Clinical Effectiveness of Nebulized Magnesium Sulfate in Pediatric Acute Asthma: A Systematic Review

**DOI:** 10.7759/cureus.107658

**Published:** 2026-04-24

**Authors:** Archit Sharma, Palangutde Katherine Dimowo, Akshay Rajeev, Sabah Shakeel Shaikh, Harnoor Kaur, Lubna Mohammed

**Affiliations:** 1 Paediatrics, Wrightington, Wigan and Leigh Teaching Hospitals NHS Foundation Trust, Wigan, GBR; 2 Otolaryngology - Head and Neck Surgery, California Institute of Behavioral Neurosciences and Psychology, California, USA; 3 Hematology, Great Western Hospital, Swindon, GBR; 4 Diabetes and Endocrinology, California Institute of Behavioral Neurosciences and Psychology, California, USA; 5 Internal Medicine, California Institute of Behavioral Neurosciences and Psychology, California, USA; 6 Principles and Practice of Clinical Research, Harvard T.H. Chan School of Public Health, Boston, USA

**Keywords:** acute asthma managment, adjunct therapy, asthma exacerbations, nebulized magnesium sulfate, pediatric asthma

## Abstract

Asthma is a common cause of acute illness and healthcare utilization in children, and while intravenous magnesium sulfate is established in severe exacerbations, the clinical role of nebulized magnesium sulfate remains uncertain. This systematic review evaluated whether nebulized magnesium sulfate, used as an adjunct to standard therapy, improves clinical outcomes in pediatric acute asthma exacerbations. A comprehensive literature search was conducted in accordance with the Preferred Reporting Items for Systematic Reviews and Meta-Analyses (PRISMA 2020) guidelines, identifying studies published between January 2010 and November 2025. Seven studies were included: six randomized controlled trials and one systematic review with meta-analysis, all involving pediatric populations. Study selection, data extraction, and quality appraisal were performed using validated tools, including A Measurement Tool to Assess Systematic Reviews (AMSTAR 2) and the Revised Cochrane Risk-of-Bias tool for randomised trials (RoB 2). Across the included studies, nebulized magnesium sulfate demonstrated variable and generally modest effects on asthma severity scores, with no consistent reduction in hospital admission rates, length of stay, or escalation of care. Safety findings were reassuring, with no serious treatment-related adverse events reported. Overall, current evidence does not demonstrate a clear or consistent additional clinical benefit of nebulized magnesium sulfate beyond standard therapy in pediatric acute asthma exacerbations. Future well-designed trials using standardized outcome measures and longer follow-up are required to clarify its role in contemporary pediatric asthma management.

## Introduction and background

Asthma and other allergic diseases are among the most commonly encountered conditions in children [1]. Globally, asthma affects approximately 339 million people, which poses a significant public health challenge [2]. Among children, it affects nearly 22 million worldwide, with acute exacerbation contributing to approximately 13,000 deaths annually and representing a major cause of morbidity and mortality [3]. An acute exacerbation of bronchial asthma is characterized by an increase in airflow obstruction in the respiratory tract. It is marked by a worsening of symptoms, such as breathlessness, cough, and chest tightness, along with a gradual increase in work of breathing and decline in lung function [4]. Early management of asthma exacerbations relies on a limited evidence-based treatment approach. First-line therapy includes bronchodilators, such as short-acting and long-acting beta-2 agonists, anticholinergic agents like ipratropium bromide, and inhaled or systemic corticosteroids. Adjunctive therapies include magnesium, intravenous bronchodilators, and methylxanthines, which are reserved for severe or refractory cases [5].

Magnesium is an essential intracellular cation that plays a key role in numerous physiological processes and has been used therapeutically in various clinical conditions, including asthma [6]. When magnesium levels are low, calcium-driven pro-inflammatory activity predominates, increasing mediator release [7]. The mechanism by which magnesium sulfate may benefit asthma patients includes inhibition of calcium influx into bronchial smooth muscle cells, resulting in smooth muscle relaxation and bronchodilation [8]. Additional pharmacological effects attributed to magnesium sulfate include reduced release of acetylcholine and histamine, as well as modulation of enzymatic activity involving adenylyl cyclase and sodium-potassium ATPase. These mechanisms may enhance the responsiveness to β₂-agonists and contribute to their role as an adjunctive therapy during acute asthma exacerbations [9].

Magnesium sulfate is currently utilized in two principal forms for the management of acute asthma: intravenous and nebulized [6]. Meta-analysis of two key trials in both adult and pediatric populations has demonstrated that the addition of intravenous magnesium sulfate to standard therapy is associated with reduced hospital admission rates and improved pulmonary function [10,11]. Based on this evidence, authors and international asthma guidelines recommend consideration of intravenous magnesium in children who do not respond adequately to initial therapy [10,12,13]. However, intravenous magnesium sulfate may increase serum magnesium levels and requires monitoring for potential toxicity, including flushing, dizziness, nausea, and vomiting [6].

By contrast, nebulized magnesium sulfate represents a non-invasive alternative that allows targeted delivery to the lower airways and may be associated with a lower risk of systemic adverse effects [14]. Despite these theoretical advantages, the clinical benefit of nebulized magnesium remains uncertain. The available evidence is limited, and the efficacy of nebulized magnesium sulfate in acute asthma has not been clearly established [15].

While the role of intravenous magnesium sulfate is well established in severe acute asthma, the effectiveness of nebulized magnesium sulfate remains uncertain, particularly in the pediatric population. Current evidence is conflicting, and routine use as an adjunct to standard therapy is not well supported. This systematic review aims to critically evaluate the efficacy and safety of nebulized magnesium sulfate as an adjunct to standard therapy in children with acute asthma exacerbations, with a specific focus on asthma severity scores, healthcare utilization (hospital admission and length of stay), escalation of care, and adverse events.

## Review

Methods

This systematic review was conducted in accordance with the Preferred Reporting Items for Systematic Research and Meta-Analyses (PRISMA) 2020 guidelines [16]. The review methods were established prior to the conduct of the review, and no significant deviations were observed.

Database and Search Strategy

A systematic literature search was undertaken across multiple databases, like PubMed (through both PubMed Medical Subject Headings-MeSH and Advanced Search), Cochrane Library, ScienceDirect, Google Scholar, and ClinicalTrials.gov. The search strategy was constructed around keywords, such as “asthma exacerbation”, “acute asthma”, “magnesium sulphate/sulfate”, “nebulised/nebulized”, and "child". Only studies published between January 1, 2010, and November 22, 2025, were considered. An overview of the databases searched, keywords used, applied filters, and the number of records identified from each source is presented in Table 1.

**Table 1:** Overview of search strategies used across various databases and number of records retrieved

Databases	Keyword	Search strategy	Filter applied	Results
PubMed Advanced	asthma exacerbation, acute asthma, magnesium sulphate, nebulised, inhaled	(((((Asthma exacerbation) AND (Acute asthma)) AND (Magnesium sulphate))	From 01/01/2010 to 22/11/2025, free full text, English, child: birth-18 years	17
PubMed MeSH	asthma exacerbation, acute asthma, magnesium sulphate	ASTHMA OR(“Asthma/drug therapy"[Mesh] OR "Asthma/therapy"[Mesh] ) ( "Asthma/drug therapy"[Mesh] OR "Asthma/therapy"[Mesh] ) AND MAGNESIUM SULPHATE OR "Magnesium Sulfate/therapeutic use"[Mesh] "Magnesium Sulfate/therapeutic use"[Mesh]	From 01/01/2010 to 22/11/2025, free full text, English, child: birth-18 years	181
Cochrane Library (Advanced)	asthma exacerbation, acute asthma, child, magnesium sulphate	acute asthma in Title Abstract Keyword AND asthma exacerbation in Title Abstract Keyword AND "magnesium sulphate" in Title Abstract Keyword AND child in Title Abstract Keyword	English, from 01/01/2010 to 22/11/2025	28
Google scholar	asthma exacerbation, acute asthma, child, magnesium sulphate, nebulised, inhaled, English	("nebulised magnesium" OR "nebulized magnesium" OR "magnesium sulphate" OR "magnesium sulfate") AND ("acute asthma" OR "asthma exacerbation" OR "status asthmaticus" OR "acute severe asthma") AND (child* OR pediatric OR paediatric OR adolescent*) AND (English)	From 2010 to 2025	1,180
Science Direct	asthma exacerbation, acute asthma, child, magnesium sulphate	acute asthma, magnesium sulphate, child	English, open access and open archives, review articles, research articles, from 2010 to 2025	167
ClinicalTrials.gov	asthma exacerbation, acute asthma, child, magnesium sulphate, nebulised, inhaled	(Acute asthma OR asthma exacerbation) AND (Magnesium sulphate OR Magnesium sulfate) AND (Child OR Children)	Completed	11

Inclusion Criteria

Studies were considered eligible for inclusion if they investigated a pediatric population with asthma, defined as children from birth to 18 years of age. Eligible study designs included established research methodologies, like randomized controlled trials, comparative observational studies, systematic reviews, and meta-analyses. In addition, studies were required to evaluate the use of inhaled or nebulized magnesium sulfate as part of the intervention and be available as a free full-text in the English language.

Exclusion Criteria

Studies were excluded if they involved adult-only populations or mixed adult-pediatric cohorts or if they focused on intravenous or oral magnesium sulfate rather than the nebulized formulations. Studies were excluded if they had no reported results, including ongoing or prematurely terminated trials, or if they were presented solely as commentaries, abstracts, study protocols, or unpublished trials. Studies conducted in animal models or in vitro settings were additionally excluded.

Selection Process

The records retrieved from various databases were imported to Rayyan AI for systematic data management and duplication removal. Results obtained from Google Scholar were first organized in Mendeley before being transferred to ensure accurate integration from all sources. After the complete consolidation of the dataset in Rayyan, titles and abstracts were screened to match the review’s objective. Studies deemed potentially relevant then underwent full-text evaluation according to the specified inclusion and exclusion standards. Two reviewers independently screened titles, abstracts, and full texts for eligibility and extracted data. After confirming eligibility, the studies meeting these criteria were subjected to quality assessment using appropriate, design-specific tools. Only studies judged to be of high quality were included in the systematic review.

*Quality Appraisal*

To ensure the robustness and credibility of evidence, each eligible study was subjected to rigorous quality analysis using tools appropriate to its design. Systematic reviews were appraised using A MeaSurement Tool to Assess Systematic Reviews version 2 (AMSTAR 2) [17]. Randomized controlled trials were evaluated using the Revised Cochrane Risk of Bias (RoB 2) tools, allowing a structured examination of potential bias along key domains, like randomization, blinding, and outcome reporting [18]. Risk of bias and methodological quality were independently assessed by two reviewers using design-specific appraisal tools.

Results

**Figure 1:**
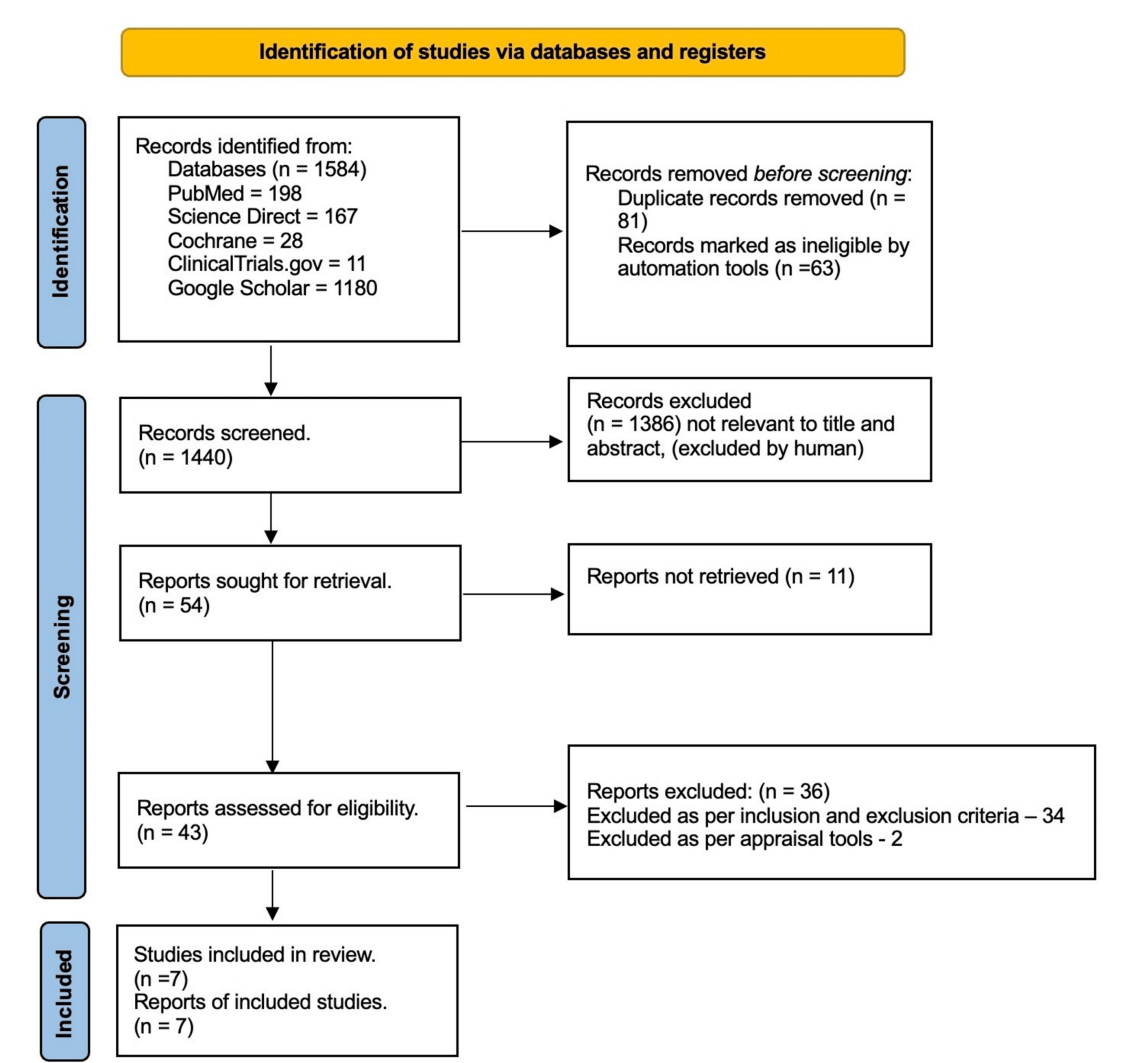
PRISMA 2020 flow diagram for systematic reviews illustrating study selection process PRISMA: Preferred Reporting Items for Systematic Research and Meta-Analyses [16]

A total of 1,584 records were identified across the databases searched (PubMed: 198; ScienceDirect: 167; Cochrane: 28; Google Scholar: 1180; ClinicalTrials.gov: 11). After removal of duplicate records, 1,440 studies remained for title and abstract screening. Following this screening, 54 studies were identified as potentially eligible and were sought for retrieval. Out of the 54 studies, 43 studies were retrieved for full-text assessment in accordance with the predefined inclusion and exclusion criteria. Of these, 34 studies did not meet the above-mentioned eligibility criteria. Nine studies were then screened for methodological quality appraisal, and seven studies met the predefined quality thresholds and were included in the final systematic review. The study selection process is summarized as a PRISMA flow diagram in Figure 1. 

The methodological quality of the included systematic review and meta-analysis was assessed using AMSTAR 2 [17]. The result of this appraisal is summarized in Table 2.

**Table 2:** Quality assessment of the included systematic review and meta-analysis using the AMSTAR 2 tool Each item was rated as *Yes*, *Partial Yes*, or *No* in line with AMSTAR 2 guidance, with *Partial Yes* applied only where permitted. Critical domains were evaluated and interpreted according to AMSTAR 2 recommendations [17].

	AMSTAR 2 domain	Kumar et al. [3]
1.	PICO clearly defined	Yes
2.	Protocol registered prior to review	Yes
3.	Rationale for study design selection	Yes
4.	Comprehensive literature search	Yes
5.	Study selection in duplicate	Yes
6.	Data extraction in duplicate	Yes
7.	Excluded studies listed with justification	Yes
8.	Adequate description of included studies	Yes
9.	Appropriate risk of bias assessment	Yes
10.	Funding sources of the included studies reported	No
11.	Appropriate meta-analysis methods	Yes
12.	Impact of RoB on results assessed	No
13.	RoB considered in interpretation	Yes
14.	Heterogeneity assessed and discussed	Yes
15.	Publication bias assessed	Yes
16.	Conflict of interest reported	Yes
		High quality

The risk of bias for the included randomized controlled trials was evaluated using the RoB 2, assessing bias across five predefined domains [18]. A summary of domain-level and overall risk-of-bias judgement is presented in Table 3.

**Table 3:** Risk-of-bias assessment of the included randomized controlled trials using the Cochrane Risk-of-Bias (RoB 2) Tool Risk of bias was assessed using the Cochrane Risk-of-Bias (RoB 2) tool across five domains. Domains 1 to 5 signify bias arising from the randomization process, bias due to deviations from intended interventions, bias due to missing outcome data, bias in measurement of the outcome, and bias in the selection of the reported result, respectively. Each domain was rated as low risk, some concerns, or high risk. Overall risk-of-bias judgments were made according to the RoB 2 guidance [18].

Study	Domain 1	Domain 2	Domain 3	Domain 4	Domain 5	Overall
Powell et al. [19]	Low risk	Low risk	Low risk	Low risk	Low risk	Low risk
Alansari et al. [20]	Low risk	Some concerns	Low risk	Low risk	Low risk	Some concerns
Daengsuwan et al. [6]	Some concerns	Some concerns	Low risk	Some concerns	Some concerns	Some concerns
Schuh at al. [21]	Low risk	Low risk	Low risk	Low risk	Low risk	Low risk
Wongwaree et al. [22]	Low risk	Low risk	Low risk	Low risk	Some concerns	Some concerns
Siddiqui et al. [4]	Low risk	Some concerns	Low risk	Low risk	Some concerns	Some concerns

Discussion

The key characteristics and principal findings of the included trials evaluating nebulized magnesium sulfate in pediatric acute asthma are summarized in Table 4. 

**Table 4:** Summary of the study characteristics and key findings from trials evaluating nebulized magnesium sulfate as an adjunct to standard therapy in pediatric acute asthma PRAM: Pediatric Respiratory Assessment Measure

Authors/year	Study design	Location	Intervention	Comparator	Asthma severity score analyzed	Primary outcome studied	Key points		
Powell et al., 2013 [19]	Randomized controlled trial	United Kingdom (UK)	Nebulized magnesium sulfate + standard therapy	Nebulized placebo + standard therapy	Yung Asthma Severity Score	Change in the asthma severity score	Nebulized magnesium sulfate produced a modest but significant improvement in asthma severity in children with acute severe asthma and was safe and cost-effective, although it did not significantly reduce hospitalization.		
Alansari et al., 2015 [20]	Randomized controlled trial	Qatar	Nebulized magnesium sulfate + standard therapy	Nebulized placebo + standard therapy	PRAM	Time to medical readiness for discharge	Nebulized magnesium sulfate led to greater improvement in PRAM scores, particularly in severe asthma, with reduced need for additional bronchodilators and no safety concerns.		
Daengsuwan et al., 2017 [6]	Comparative randomized study	Thailand	Nebulized magnesium sulfate	Intravenous magnesium sulfate	Modified Wood’s clinical asthma score	Change in severity score	Nebulized magnesium sulfate showed comparable efficacy and safety to intravenous magnesium, suggesting a less invasive alternative in severe acute asthma.		
Schuh et al., 2020 [21]	Randomized controlled trial	Canada	Nebulized magnesium sulfate + albuterol	Placebo + albuterol	PRAM	Hospitalization	In children with refractory acute asthma, nebulized magnesium sulfate did not reduce hospitalization rates.		
								Wongwaree et al., 2022 [22]	Randomized controlled trial	Thailand	Nebulized magnesium sulfate	Nebulized ipratropium bromide/fenoterol	PRAM	Improvement in severity score	Nebulized magnesium sulfate was similarly effective to ipratropium bromide/fenoterol in improving asthma severity in moderate to severe exacerbations.		
								Siddiqui et al., 2022 [4]	Randomized controlled trial	India	Nebulized magnesium sulfate + salbutamol	Salbutamol alone	No validated asthma severity score used	Improvement in physiological parameters	Addition of nebulized magnesium sulfate to salbutamol resulted in faster clinical improvement, fewer repeat nebulisation, and shorter hospital stays with good tolerability.		
Kumar et al., 2024 [3]	Systematic review and meta-analysis	India	Nebulized magnesium sulfate (from pooled studies)	Placebo/standard therapy	Various (Yung, PRAM, and Pulmonary Score)	Change in clinical severity	Meta-analysis demonstrated a modest overall benefit of nebulized magnesium, particularly in severe pediatric asthma, with significant heterogeneity but a favorable safety profile.		

Current Guideline Recommendations for Magnesium Sulfate in Pediatric Acute Asthma

International guidelines consistently reserve magnesium sulfate for severe acute asthma exacerbations unresponsive to initial standard therapy [13,23,24]. The United Kingdom British Thoracic Society (BTS)/Scottish Intercollegiate Guidelines Network (SIGN 158) uniquely incorporates nebulized magnesium sulfate into early acute management for a defined pediatric subgroup, recommending 150 mg magnesium sulfate added to each nebulized salbutamol and ipratropium dose during the first hour in children with acute severe asthma and oxygen saturation <92% [13]. By contrast, the United States National Asthma Education and Prevention Program (NAEPP) Expert Panel Report 3 (EPR-3) guideline does not recommend nebulized magnesium sulfate, instead supporting selective intravenous magnesium sulfate for children with life-threatening exacerbations or persistent severe obstruction after one hour of intensive conventional therapy [23].

**Figure 2:**
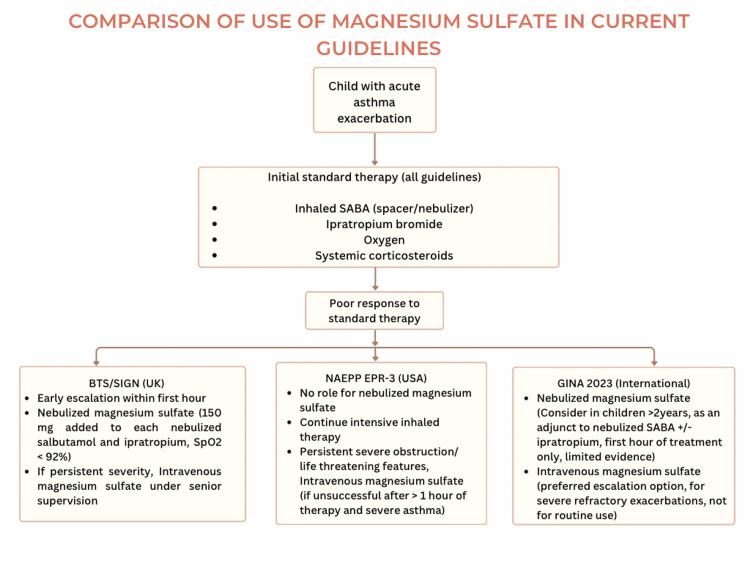
Comparison of three current guidelines on magnesium sulfate use in acute asthma exacerbation in children SABA: short-acting beta agonist. Created by the first author based on data from [13,23,24]. This figure was created by first author using Canva (Canva Pty Ltd., Australia) based on data from [13,23,24].

The Global Initiative for Asthma 2023 (GINA 2023) similarly reserves magnesium sulfate for severe diseases but explicitly allows consideration of nebulized magnesium sulfate in children aged ≥2 years as an adjuvant during the initial management of acute severe asthma, particularly in the presence of hypoxemia. However, the GINA emphasizes the limited and inconsistent evidence supporting nebulized administration and continues to favor intravenous magnesium sulfate when escalation is required [24]. These guideline discrepancies highlight ongoing clinical uncertainty and reinforce the need for systematic evaluation of nebulized magnesium sulfate as an adjunct to standard therapy in pediatric acute asthma exacerbations. A comparison of international guideline recommendations regarding the use of magnesium sulfate in pediatric acute asthma is illustrated in Figure 2.

*Effect of Nebulized Magnesium Sulfate on Asthma Severity Scores*

Asthma severity scores integrate multiple bedside clinical observations into a single numerical value, enabling clinicians to assess disease severity, guide treatment decisions, and monitor response to therapy over time [25]. Several pediatric asthma severity scoring systems are described in the literature, including the Pulmonary Score, Pediatric Respiratory Assessment Measure (PRAM), Pediatric Asthma Score (PAS), Pediatric Asthma Severity Score (PASS), Clinical Asthma Score, and Modified Wood’s Clinical Asthma Score [25].

In this context, the included randomized controlled trials have consistently demonstrated that although asthma severity improves over time with standard therapy, the addition of nebulized magnesium sulfate does not result in a clinically meaningful further reduction in severity [20,21]. This has been shown using validated tools such as the PRAM score in children with moderate-to-severe and severe asthma exacerbations [20,21]. These neutral findings are further reinforced by Kumar et al., whose pooled analysis combining multiple severity instruments, including the PRAM, Asthma Severity Score, and Modified Pulmonary Index Score, demonstrated a negligible standardized mean difference and no clinically relevant benefit of nebulized magnesium sulfate [3].

By contrast, the Magnesium Nebuliser Trial in Children (MAGNETIC Trial), which employed the Yung Asthma Severity Score, reported a statistically significant but modest improvement following nebulized magnesium sulfate, with a reduction observed at 60 minutes and persisting up to four hours [19]. However, the magnitude of benefit was explicitly described as clinically marginal, and the clinical relevance of these findings therefore remains uncertain [19]. Similarly, other studies using the Modified Wood’s Clinical Asthma Score or the PRAM score found that nebulized magnesium was not superior to other treatments such as intravenous magnesium sulfate or ipratropium bromide/fenoterol. Both groups showed similar improvements, with no significant differences between them [6,22].

Siddiqui et al. did not use a validated clinical asthma severity score and instead relied on physiological measures such as the peak expiratory flow rate (PEFR), respiratory rate, and oxygen saturation as proxies for severity. In this Indian randomized controlled trial comparing nebulized salbutamol with or without magnesium sulfate, similar improvements in PEFR and vital signs were observed, but no between-group differences were demonstrated, supporting a neutral effect on clinical severity [4]. Taken together, the available evidence shows inconsistent and generally modest effects of nebulized magnesium sulfate on asthma severity beyond standard therapy and should therefore be interpreted with caution.

Healthcare Utilization and Escalation of Care Outcomes

Across randomized controlled trials evaluating nebulized magnesium sulfate as an adjunct to standard therapy in children with acute asthma exacerbations, no consistent benefit was observed in hospital admission-related outcomes [3,19,21]. Studies comparing nebulized magnesium sulfate with placebo or active comparators reported no significant reduction in initial hospitalization rates, including in large trials enrolling children with moderate to severe exacerbations [3,19,21].

Similarly, among the included studies, the length of hospital stays and time to medical readiness for discharge were not markedly shortened in children receiving nebulized magnesium sulfate, despite adequate power and rigorous trial design [3,19-21]. Analysis of early discharge readiness within 24 hours demonstrated no meaningful differences between intervention and control groups, with confidence intervals persistently overlapping the null [3,19,20]. Collectively, these findings indicate that nebulized magnesium sulfate does not influence admission decisions, inpatient duration, or discharge timing when added to standard treatment in pediatric populations [3,19-21].

In parallel, escalation-of-care outcomes, such as pediatric intensive care unit admissions, mechanical ventilation, or need for higher-level respiratory support, were comparable between intervention and control groups, even among children presenting with severe asthma exacerbations [3,6,19-21]. Trials assessing treatment progression found that nebulized magnesium sulfate did not reduce the subsequent need for intravenous magnesium sulfate or bronchodilators, suggesting no apparent prevention of escalation to parenteral escalation, when the initial treatment failed [6,19,21,22].

Furthermore, post-discharge healthcare utilization, including emergency department revisits, readmissions, and health service use following discharge, was similar between intervention and control groups [19-21]. Despite heterogeneity in study designs, comparators, and severity thresholds, the direction of effect across trials was consistent, demonstrating no clinically important improvement in patient-centered or health-system outcomes with nebulized magnesium sulfate as an adjunct in pediatric acute asthma exacerbations [3,6,19-22].

Safety and Tolerability of Nebulized Magnesium Sulfate

The inhaled route of magnesium sulfate provides a non-invasive means of drug delivery with rapid onset of action and limited systemic exposure, which may be associated with a more favorable safety profile [26]. Across the evidence base, magnesium sulfate appears to be well tolerated in the short term, with no serious treatment -attributable adverse events reported in included studies [4,6,19-22]. In the largest emergency department-based trial, adverse events were infrequent and mostly mild, occurring more often in the magnesium group and consisting of nausea, vomiting, and upper airway irritation. However, serious adverse events were limited to asthma-related ICU admissions and were not attributable to nebulized magnesium, with no observed increase in clinically significant hypotension or apnea [21]. Likewise, the MAGnesium NEbuliser Trial In Children (MAGNETIC) trial reported comparable adverse event rates between magnesium and placebo groups, with events described as mild and transient, while a large inpatient randomized controlled trial found no hypotension, largely comparable vital signs, and only isolated self-limiting reactions in both study groups [19,20].

Comparative studies demonstrated a reassuring safety profile: the trial comparing nebulized with intravenous magnesium reported no adverse effects in either group despite higher serum magnesium levels with intravenous administration [6], while the head-to-head study against ipratropium/fenoterol reported only minor self-limiting symptoms with nebulized magnesium [22]. In the Indian randomized controlled trial, no clinically reported adverse effects or signs of magnesium toxicity were observed despite active monitoring [4]. Consistent with this, pooled analysis by Kumar et al. confirmed that adverse events were infrequent and generally minor, with no clear increase in overall risk, although certainty was limited by low event rates and variable reporting [3]. However, all included studies evaluated safety over short follow-up periods, ranging from hours to a few days [4,6,19,21,22], which limits the ability to detect rare, delayed, or cumulative adverse effects. Therefore, available evidence is insufficient to draw conclusions regarding rare or delayed adverse effects. A consolidated summary of efficacy outcomes, healthcare utilization, escalation of care, and safety findings across the included studies is provided in Table 5.

**Table 5:** Summary of outcomes reported in included studies evaluating nebulized magnesium sulfate as an adjunct to standard therapy in pediatric acute asthma.

Study	Impact on the asthma severity score	Hospitalization/length of stay (LOS)	Escalation of care/PICU admission	Post-discharge resource utilization – ED revisits or readmission	Safety and adverse events
Powell et al. [19]	Statistically significant but clinically marginal in the Yung Asthma Severity Score	No reduction	No difference	No difference	Mild and transient, adverse event rates, similar between groups
Alansari et al. [20]	No clinically meaningful difference in PRAM score improvement	No reduction	No difference	No difference	No serious adverse events
Daengsuwan et al. [6]	Non-inferior, improvement in the Modified Wood’s Clinical Asthma Score	No difference	No reduction	Not reported	No adverse effect in either group; high serum magnesium with IV magnesium
Schuh et al. [21]	No additional benefit on PRAM score	No reduction	No difference	No difference	Mild adverse events
Wongwaree et al. [22]	No superiority, parallel improvement in PRAM score	No difference	No difference	Not reported	Minor, self-limiting
Siddiqui et al. [4]	No validated severity score use, significant within-group improvement but no between-group difference in physiological parameters	No difference	Not reported	Not reported	No adverse events
Kumar et al. [3]	Negligible standardised mean difference across PRAM, Asthma Severity Score, and Modified Pulmonary Index score	No reduction	No reduction	No difference	Infrequent and low

Limitations

Asthma severity was assessed using multiple clinical scoring systems, including PRAM, Yung Asthma Severity Score, Modified Wood’s Clinical Asthma Score, and other composite indices, while one study relied on physiological parameters rather than a severity score, limiting direct comparability and synthesis of efficacy outcomes. In addition, the comparator interventions varied considerably, with nebulized magnesium sulfate evaluated against placebo, standard therapy alone, intravenous magnesium sulfate or alternative bronchodilator regimes, introducing indirectness and complicating interpretation of its benefit as an adjunct treatment. These sources of heterogeneity reduce confidence in pooled estimates and necessitate cautious interpretation of the findings. Interpretation of safety findings is further constrained by short follow-up periods, variability in adverse event definition and reporting, and biochemical monitoring inconsistently performed. Consequently, conclusions reflect short-term tolerability rather than rare or delayed adverse effects and should therefore be drawn with caution.

## Conclusions

This systematic review demonstrates that nebulized magnesium sulfate does not provide a consistent, meaningful additional benefit when used as an adjunct to standard therapy in pediatric acute asthma exacerbations. Across the included studies, improvements in asthma severity scores were generally modest and frequently failed to reach clinical significance, with no clear reduction in hospital admission rates, length of stay, or escalation of care. Nebulized magnesium sulfate was well tolerated in the short term, with no serious treatment-related adverse events reported across studies. However, safety data were limited to brief follow-up periods, and conclusions regarding rare or delayed adverse effects cannot be drawn. Overall, the current evidence does not support routine use of nebulized magnesium sulfate in pediatric acute asthma management. This aligns with existing guideline recommendations, which reserve magnesium sulfate for selected children with severe exacerbations who fail to respond to initial therapy. Future research should focus on adequately powered, well-designed trials using standardized severity scoring systems, clearly defined clinical outcomes, and longer follow-up to determine whether specific subgroups, such as children with severe or refractory exacerbations, may derive meaningful benefit.
